# LncRNA-driven programmed cell death networks: new therapeutic targets for neurological disorders

**DOI:** 10.3389/fnmol.2025.1635119

**Published:** 2025-07-24

**Authors:** Zihong Xiong, Chao Sun, Shiyong Huang

**Affiliations:** ^1^Department of Pediatric Intensive Care Unit, Chengdu Women’s and Children’s Central Hospital, School of Medicine, University of Electronic Science and Technology of China, Chengdu, China; ^2^Department of Pediatrics, Chongzhou Health Center for Women and Children, Chongzhou, China; ^3^Department of Pediatrics, Southwest Medical University, Luzhou, China

**Keywords:** long non-coding RNA, central nervous system disease, neural cells, programmed cell death, cell death

## Abstract

Neural cell death is a critical pathological mechanism underlying the development and progression of central nervous system (CNS) diseases, where programmed cell death (PCD) pathways serve as critical regulatory hubs. In addition to classical apoptosis and autophagy, emerging PCD modalities including necroptosis, pyroptosis, ferroptosis, and cuproptosis exhibit distinct activation patterns in different neurological diseases. Long non-coding RNAs (lncRNAs) have emerged as pivotal regulators of these PCD processes through multiple molecular strategies by modulating chromatin accessibility, assembling signaling complexes, and regulating post-transcriptional processes. These regulatory interactions vary by cellular location and disease stage, influencing cell fate through membrane receptors, kinase cascades, and nuclear transcriptional programs. In various CNS pathologies, specific lncRNAs display dual regulatory capacities—promoting neuronal death by amplifying cytotoxic signals or conferring neuroprotection by inhibiting these pathways. The dynamic lncRNA-PCD interactions offer therapeutic potential through targeted modulation of lncRNA networks to control neuronal survival. Future investigations should prioritize systematic mapping of context-specific lncRNA regulatory networks governing distinct PCD modalities, concurrently advancing spatial epigenomic editing technologies for precise manipulation of these regulatory circuits. Understanding these molecular interactions better will help identify therapeutic targets and guide CNS drug development.

## 1 Introduction

Long non-coding RNAs (lncRNAs), defined as longer than 200 nucleotidest non-protein-coding transcripts (Nitsche and Stadler, 2017), have transitioned from being mischaracterized as transcriptional noise to emerging as master regulators of gene expression through diverse mechanisms including transcriptional modulation (histone modification, cofactor recruitment), RNA processing (alternative splicing), and post-translational regulation—notably functioning as endogenous RNA sponges within competing endogenous RNA (ceRNA) networks (Khoshnam et al., 2024; Kitagawa et al., 2013; Ruffo et al., 2023; Wu et al., 2021; Yang et al., 2023). Their region-specifically enriched expression in murine hippocampal, cortical, and thalamic structures (Kadakkuzha et al., 2015) and zebrafish CNS (brain/spinal cord) (Kaushik et al., 2013) demonstrates conserved regulation across vertebrate neural systems, underpinning roles in coordinating neural cell proliferation, differentiation, and programmed death (Ghafouri-Fard et al., 2022; Herman et al., 2022; Kleaveland et al., 2018; Wu and Kuo, 2020). This review synthesizes lncRNA-mediated regulation of neural programmed cell death (PCD) by integrating three fundamental dimensions: (1) functional convergence and divergence across apoptosis, autophagy, pyroptosis, ferroptosis, and cuproptosis pathways; (2) shared mechanistic principles spanning ceRNA networks and epigenetic reprogramming; and (3) context-specific dysregulation in neurodegeneration and neurotrauma, thereby addressing the identified knowledge gap in lncRNA-PCD interactomes within the nervous system.

## 2 LncRNA-mediated programmed neural cell death in central nervous system diseases

### 2.1 Apoptosis

Apoptosis—an evolutionarily conserved cell death mechanism mediating tissue homeostasis—executes through cytoplasmic shrinkage, nuclear fragmentation, and apoptotic body formation via two core pathways: (1) The mitochondrial pathway, where oxidative stress induces Bax/Bak-mediated mitochondrial pore formation, cytochrome c release, and apoptosome assembly (Apaf-1/pro-caspase-9), culminating in caspase-9 autoactivation and downstream caspase-3/7 proteolysis; (2) The death receptor pathway, initiated by extracellular ligands (FasL/TNF-ɑ) that trimerize receptors to recruit FADD and pro-caspase-8, forming the DISK complex for caspase-8 dimerization-driven autocleavage (Amgalan et al., 2017; Fernández et al., 2015; Riley and Bock, 2022; Schwarzer et al., 2020; Van Opdenbosch and Lamkanfi, 2019). Crucially, while dysregulation of these pathways drives neurodegeneration in cerebral ischemia and chronic disorders, lncRNAs’ regulatory roles in modulating key molecular switches—such as Bax activation thresholds, apoptosome stoichiometry, or DISK assembly kinetics—remain underexplored in neural contexts.

Emerging evidence establishes specific lncRNAs as critical coordinators of cell fate across pathologies. In solid tumors, plasmacytoma variant translocation 1 (PVT1) and p21-associated lncRNA exemplify dual-function regulators orchestrating cellular apoptosis while mediating therapeutic resistance through apoptotic threshold modulation (Ferrer and Dimitrova, 2024; He et al., 2018; Li et al., 2022). In ischemic stroke, MEG3 orchestrates bimodal apoptotic control via hnRNPA1 sequestration to induce Sirt2-dependent mitochondrial dysfunction (Yao et al., 2024), concurrently sequestering cytoplasmic miR-21 to elevate PDCD4 and activate caspase-3 (Yan et al., 2017). The lncRNA KCNQ1OT1 exerts neurotoxic effects by competitively binding miR-296-5p, upregulating Bax expression and enhancing mitochondrial cytochrome c release (Li et al., 2020), while SNHG14 attenuates neuronal injury via miR-98-5p sponging to upregulate BCL2L13 in neuro-2a cells (Zhang et al., 2022). Functional heterogeneity is evident in NEAT1: Global suppression dysregulates synaptic plasticity (Li and Wang, 2023), whereas pharmacological induction by bexarotene elevates NEAT1 to attenuate Pidd1-caspase-2 signaling in traumatic brain injury (Zhong et al., 2017). Neuroprotective mechanisms include N1LR-mediated inhibition of p53 phosphorylation at Ser15 (Wu et al., 2017), and TUG1 promoting neuronal apoptosis through miR-9 sponging and Bcl2l11 upregulation (Chen et al., 2017). The ZFAS1 neuroprotective axis demonstrates miR-582 sequestration to upregulate NOS3 expression (Zhang and Zhang, 2020), while Rian attenuates apoptosis via miR-144-3p/GATA3 signaling (Yao et al., 2020) and Oprm1 reduces cell death through miR-155/GATA3 signaling (Jing et al., 2019). Critically, comparative profiling reveals divergent targeting of death pathways: stroke-associated lncRNAs (MEG3, KCNQ1OT1) converge on mitochondrial apoptosis, whereas neuroprotective lncRNAs (NEAT1, ZFAS1, N1LR) modulate caspase-dependent survival. These systematically cataloged mechanisms establish lncRNAs as hierarchically organized master regulators of neuronal fate (Table 1).

**TABLE 1:** Long non-coding RNAs (LncRNA)-driven neuronal apoptosis: targets and mechanisms.

LncRNA	Disease model	Expression	Molecular axis	Functional outcome	References
MEG3	Ischemic stroke	↑	MEG3↑→ miR-21↓→ PDCD4↑ MEG3↑→ hnRNPA1↓→ Sirt2↓	Drives neuronal death via PDCD4 signaling and mitochondrial dysfunction	Yan et al., 2017; Yao et al., 2024
KCNQ1OT1	Neuroblastoma	↑	KCNQ1OT1↑→ miR-296-5p↓→ Bax↑	Promotes apoptosis via Bax activation	Li et al., 2020
SNHG14	OGD/R neuro-2a model	↑	SNHG14↑→ miR-98-5p↓→ BCL2L13↑	Reduces apoptosis in neuronal cells	Zhang et al., 2022
NEAT1	Traumatic brain injury	↑	Bexarotene↑→ NEAT1↑→ PIDD1↓→ Caspase-2↓	Bexarotene-induced neuroprotection	Li and Wang, 2023; Zhong et al., 2017
N1LR	Ischemic stroke	↓	N1LR overexpression → p53-Ser15 phosphorylation↓→ PUMA↓	Neuroprotection via p53 inhibition in neurons	Wu et al., 2017
TUG1	OGD-induced neurons	↑	TUG1↑→ miR-9↓→ Bcl2l11↑	Promotes neuronal apoptosis via Bim activation	Chen et al., 2017
ZFAS1	Cerebral I/R injury	↑	ZFAS1↑→ miR-582↓→ NOS3↑	Neuroprotection via NOS3-mediated apoptosis inhibition	Zhang and Zhang, 2020

OGD/R, oxygen-glucose deprivation/reperfusion; I/R, ischemia/reperfusion; PDCD4, programmed cell death 4; hnRNPA1, heterogeneous nuclear ribonucleoprotein A1; Sirt2, sirtuin 2; Bax, BCL2-associated X protein; BCL2L13, BCL2 like 13; PIDD1, p53-induced death domain protein 1; PUMA, p53 upregulated modulator of apoptosis; Bim, BCL2-like 11 (Bcl2l11); NOS3, nitric oxide synthase 3;↑, upregulation; ↓, downregulation.

### 2.2 Autophagy

Autophagy—an evolutionarily conserved lysosomal degradation system comprising three mechanistically distinct forms: microautophagy, macroautophagy, and chaperone-mediated autophagy (Dikic and Elazar, 2018)—facilitates clearance of damaged organelles and protein aggregates through double-membrane autophagosome formation. This catabolic process serves dual homeostatic and stress-responsive functions against oxidative injury, ischemia, and infection (Bernard et al., 2015; Lei and Klionsky, 2021; Mizushima et al., 2008; Siddiqui et al., 2015), governed by the PI3K-AKT1-mTOR signaling axis and core regulators (mTOR, Beclin-1, ATGs, AMPK). Crucially, while macroautophagy plays a defined pathogenic role in neurodegeneration through mediating aberrant protein clearance failure (Nixon, 2013), lncRNAs’ regulatory involvement in modulating these molecular components—particularly through PI3K-AKT1-mTOR pathway interaction—remains uncharacterized in neurological contexts.

Dysfunctional autophagy drives proteinopathic neurodegeneration across CNS disorders: Glial autophagic failure exacerbates amyloidopathy and tauopathy in Alzheimer’s disease (Litwiniuk et al., 2023), while mutant ataxin-3-induced autophagic flux disruption in Machado-Joseph disease (SCA3) is mitigated by Beclin-1 enhancement (Nascimento-Ferreira et al., 2011). LncRNAs regulate neuronal homeostasis via competitive endogenous RNA (ceRNA) mechanisms, exemplified by H19’s context-dependent duality: In adult cerebral ischemia, it sequesters miR-29a to activate ERK1/2-dependent autophagy via DUSP5 suppression, exacerbating injury (Wang et al., 2017); in neonatal hypoxic-ischemic encephalopathy, it sponges miR-29b to promote AKT3-mediated mTOR inhibition, reducing neuronal apoptosis (Zhu et al., 2022). LncRNA-driven PCD networks in Parkinson’s disease demonstrate complementary control: Downregulated SNHG1 activates autophagy via miR-221/222-p27-mTOR signaling (Qian et al., 2019), while BDNF-AS upregulation promotes autophagic apoptosis through miR-125b-5p suppression (Fan et al., 2020), concurrent with NEAT1 stabilizing PINK1 to reinforce autophagic clearance (Yan et al., 2018). This functional dichotomy—SNHG1 impeding neuronal death versus BDNF-AS accelerating degeneration—epitomizes context-dependent lncRNA governance within MPTP-induced Parkinsonian models. In cerebral ischemia, C2dat2 sequesters miR-30d-5p to activate DDIT4-dependent autophagy and apoptosis, potentiating reperfusion injury (Xu et al., 2021), while SNHG12 silencing amplifies mesenchymal stem cell efficacy by activating PI3K/AKT/mTOR signaling (Li et al., 2019). MALAT1 orchestrates microvascular protection through validated complementary routes: Sequestering miR-200c-3p in oxygen-glucose deprivation to trigger SIRT1-mediated survival (Wang et al., 2019), and sponging miR-26b during hypoxia-reoxygenation to enhance ULK2-dependent cytoprotection (Li et al., 2017). Therapeutically, Lethe enhances autophagic clearance in cortical neurons to mitigate sepsis-induced brain injury (Mai et al., 2019), while Cox2 knockdown suppresses NLRP3 inflammasome activation via coordinated autophagy regulation (Xue et al., 2019). This plasticity—H19’s duality and MALAT1’s microenvironment-specific outcomes—confirms lncRNAs as master integrators of programmed cell death. These programmable circuits provide transformative therapeutic strategies, with regulatory hierarchies mapped in Table 2.

**TABLE 2:** Long non-coding RNAs (LncRNA)-driven neuronal autophagy: targets and mechanisms.

LncRNA	Disease model	Expression	Molecular axis	Functional outcome	References
H19	Cerebral ischemia/reperfusion	↑	H19↑→ mTOR↓→ autophagy↑	Induces neuronal injury via mTOR-dependent autophagy	Wang et al., 2017
H19	Neonatal HIE	↑	H19↑→ miR-29b↓→ PI3K/AKT↑→ mTOR↑	Reduces neuroprotective autophagy via mTOR activation	Zhu et al., 2022
SNHG1	Parkinson’s disease MPTP-treated SH-SY5Y	↓	SNHG1↓→ miR-221↓/miR-222↓→ p27↑→ mTOR↓	Enhances neuroprotective autophagy and prevents cell death	Qian et al., 2019
BDNF-AS	MPTP-induced Parkinson’s disease	↓	BDNF-AS↓→ miR-125b-5p↑→ BCL2↓→ ULK1↑	Enhances neuroprotective autophagy and promotes apoptosis	Fan et al., 2020
NEAT1	MPTP-induced Parkinson’s disease	↑	NEAT1↑→ TRIM11↑→ PARIS↓→ PINK1↑	Promotes protective mitophagy by stabilizing PINK1 protein	Yan et al., 2018
C2dat2	Cerebral ischemia-reperfusion	↑	C2dat2↑→ miR-30d-5p↓→ DDIT4↑→ mTOR↓	Promotes neuronal apoptosis and autophagy via the miR-30d-5p/DDIT4/mTOR axis	Xu et al., 2021
SNHG12	Cerebral ischemia/reperfusion	↑	SNHG12↓→ IGF-1↑→ PI3K/AKT/mTOR↑	Enhances MSC therapy efficacy by suppressing neuronal apoptosis and autophagy	Li et al., 2019
MALAT1	OGD-treated brain microvascular endothelial cells	↑	MALAT1↑⟷ miR-200c-3p↓→ SIRT1↑	Protects against OGD injury via SIRT1-mediated pro-survival autophagy	Wang et al., 2019
MALAT1	OGD/R-treated brain microvascular endothelial cells	↑	MALAT1↑→ miR-26b↓→ ULK2↑	Protects against OGD/R injury by inducing pro-survival autophagy	Li et al., 2017
Lethe	CLP-induced sepsis brain injury	↑	Lethe↑→ FoxO3a nuclear translocation↑→ Autophagy genes↑	Attenuates neuronal apoptosis via enhancing protective autophagy flux	Mai et al., 2019
Cox2	Experimental Autoimmune Encephalomyelitis	↑	Cox2↑→ hnRNPA2B1↑→ NLRP3 inflammasome↑→ autophagy↓	Amplifies neuroinflammation by activating NLRP3 inflammasome and suppressing autophagy	Xue et al., 2019

HIE, hypoxic-ischemic encephalopathy; MPTP, 1-methyl-4-phenyl-1,2,3,6-tetrahydropyridine; OGD, oxygen-glucose deprivation; OGD/R, oxygen-glucose deprivation/reperfusion; CLP, cecal ligation and puncture; SH-SY5Y, human neuroblastoma cell line; mTOR, mechanistic target of rapamycin; PI3K, phosphoinositide 3-kinase; AKT, protein kinase B; BCL2, B-cell lymphoma 2; ULK1, unc-51 like autophagy activating kinase 1; TRIM11, tripartite motif-containing 11; PARIS, PARK2-interacting substrate; PINK1, PTEN induced kinase 1; DDIT4, DNA damage inducible transcript 4; IGF-1, insulin-like growth factor 1; MSC, mesenchymal stem cells; SIRT1, sirtuin 1; FoxO3a, forkhead box O3; NLRP3, NLR family pyrin domain containing 3; hnRNPA2B1, heterogeneous nuclear ribonucleoprotein A2/B1; ↑, upregulation; ↓, downregulation.

### 2.3 Pyroptosis

Pyroptosis represents a caspase-dependent lytic cell death pathway characterized by gasdermin-mediated membrane pore formation and proinflammatory cytokine release. Its activation occurs through two distinct routes: the canonical pathway triggered by NOD-like receptor inflammasomes (NLRP3, NLRC4, AIM2, Pyrin) activating caspase-1, and the non-canonical pathway dependent on caspase-4/5/11 (Ai et al., 2024; Bai and Zhang, 2021; Huang et al., 2021; Mi et al., 2022; Paerewijck and Lamkanfi, 2022; Pang and Vince, 2023). Both pathways converge on gasdermin-D (GSDMD) proteolysis, where liberated N-terminal fragments oligomerize to form membrane pores enabling IL-1β/IL-18 maturation and secretion—culminating in inflammatory cell lysis. Crucially, regulatory lncRNAs’ roles in modulating sensor activation thresholds or GSDMD processing remain uncharacterized in neurological contexts. Aberrant pyroptosis drives neuroinflammatory pathogenesis in Alzheimer’s, ischemic stroke, epilepsy, and Parkinson’s disease through persistent cytokine dysregulation and neuronal degeneration (Christgen and Kanneganti, 2020; Lünemann et al., 2021; Yan et al., 2022), positioning this pathway as a pathological driver and therapeutic target whose lncRNA regulatory components warrant investigation.

Emerging evidence establishes lncRNAs as master regulators of pyroptotic networks through spatiotemporally precise, cell-type-specific mechanisms. In microglia, Parkinson’s-associated SNHG1 amplifies pyroptosis via the miR-7/NLRP3 axis (Cao et al., 2018; Zhou et al., 2016), spinal injury-induced F630028O10Rik hijacks TLR4/PI3K/AKT signaling to trigger inflammasome activation (Xu et al., 2020), and diabetic hyperglycemia elevates Fendrr expression to stabilize NLRC4 through HERC2-mediated anti-ubiquitination (Wang L. Q. et al., 2021). Conversely in neurons, ischemia-activated H19 drives NLRP3/NLRP6 inflammasomes via the miR-21/PDCD4 ceRNA network (Wan et al., 2020), while hypoxia-induced MEG3 assembles AIM2 inflammasomes to activate caspase-1 cascades (Liang et al., 2020). Neurovascular dysfunction involves endothelial Xist promoting NLRP3-dependent barrier disruption via pyroptotic cleavage (Guo et al., 2022). Therapeutically, hyperbaric oxygen (HBO) suppresses H19/miR-423-5p/NLRP3 in neural stem cells to alleviate pyroptosis (Ye et al., 2022), whereas protocatechuic aldehyde inhibits Xist/NLRP3 signaling to preserve vascular integrity (Guo et al., 2022)—notably, H19 concurrently initiates microglial pyroptosis propagating neuronal deat (Wan et al., 2020). Collectively, lncRNAs architect compartmentalized neuroinflammation by coordinating microglial inflammasome priming (SNHG1, F630028O10Rik, Fendrr), neuronal pyroptosis execution (H19, MEG3), and endothelial barrier disruption (Xist), establishing a multi-tiered druggable architecture mapped in Table 3.

**TABLE 3:** Long non-coding RNAs (LncRNA)-driven neuronal pyroptosis: targets and mechanisms.

LncRNA	Disease model	Expression	Regulatory axis	Functional outcome	References
SNHG1	Parkinson’s disease	↑	Sponges miR-7 → Activates NLRP3	Promotes microglial pyroptosis and neuroinflammation	Cao et al., 2018
F630028O-10Rik	Spinal cord injury	↑	Firre↑→↓miR-1231-5p →↑Col1a1 Firre↑→ TLR4↑→ PI3K/AKT↑	Initiates TLR4/PI3K/AKT-dependent microglial pyroptosis	Xu et al., 2020
Fendrr	Diabetic cerebral I/R injure	↑	Fendrr↑→ HERC2 binding →↓NLRC4 ubiquitination → NLRC4↑	Stabilizes NLRC4 inflammasome to drive microglial pyroptosis	Wang L. Q. et al., 2021
H19	Retinal I/R injury	↑	H19↑→ miR-21↓→ PDCD4↑ H19↑→ NLRP3↑/NLRP6↓	Triggers microglial pyroptosis via NLRP3/NLRP6 inflammasome imbalance and neuronal death	Wan et al., 2020
MEG3	Ischemic stroke MCAO/R model	↑	MEG3↑→ miR-485↓→ AIM2↑	Induces neuronal pyroptosis via AIM2 inflammasome activation	Liang et al., 2020
Xist	Ischemic stroke	↑	Xist↑→ NLRP3 inflammasome activation	Drives cerebral microvascular endothelial cell pyroptosis	Guo et al., 2022
H19	Ischemic stroke	↑	H19↑→↓miR-423-5p → NLRP3↑	Drives neural stem cell pyroptosis via NLRP3 inflammasome activation	Ye et al., 2022

I/R, ischemia/reperfusion; MCAO/R, middle cerebral artery occlusion/reperfusion; miR, microRNA; NLRP3, NLR family pyrin domain containing 3; TLR4, Toll-like receptor 4; PI3K, phosphoinositide 3-kinase; AKT, protein kinase B; HERC2, HECT and RLD domain containing E3 ubiquitin protein ligase 2; NLRC4, NLR family CARD domain containing 4; PDCD4, programmed cell death 4; NLRP6, NLR family pyrin domain containing 6; AIM2, absent in melanoma 2; Col1a1, collagen type I alpha 1 chain; ↑,upregulation; ↓, downregulation.

### 2.4 Ferroptosis

Ferroptosis—an iron-dependent necrotic process initiated by lipid peroxidation and redox-active iron accumulation (Dixon et al., 2012)—induces oxidative destruction of polyunsaturated fatty acid-rich phospholipid membranes. Its core machinery involves Fe^2+^-catalyzed Fenton reactions propagating oxidative damage (Jiang et al., 2021), with distinctive mitochondrial ultrastructural changes differentiating it from other death modalities (Zheng and Conrad, 2020). This regulated cell death pathway integrates four interdependent metabolic axes: glutathione-dependent redox balance (Dixon et al., 2012), systemic iron homeostasis (Yang et al., 2016), lipid/amino acid metabolism (Dixon et al., 2015), and mitochondrial dynamics (Gao et al., 2019). Although hepatocarcinoma studies demonstrate GSTZ1-mediated ferroptosis sensitization via NRF2/GPX4 axis suppression (Wang Q. et al., 2021), pathological iron dysregulation predominantly drives neurological degeneration, manifesting as post-traumatic epileptogenesis through astrocyte ferroptosis (Chen et al., 2020) and Alzheimer’s progression potentiated by Aβ-NRF2 axis collapse (Lane et al., 2021). Execution converges on three druggable effector hubs—glutathione peroxidase 4 (GPX4) inactivation enabling lipid peroxidation propagation (Yang et al., 2014), System Xc^–^ dysfunction depleting glutathione reserves (Dixon et al., 2012), and p53-mediated iron metabolism reprogramming (Jiang et al., 2015)—that demonstrate epigenetic susceptibility in cancer models, designating them as priority nodes for lncRNA-driven control in neurodegeneration. Nevertheless, their disease-specific regulatory architecture—particularly lncRNA-calibrated iron homeostasis dynamics—remains uncharted, constituting a fundamental knowledge gap obstructing precision-targeted neurological therapeutics development.

Long non-coding RNAs emerge as critical ferroptosis regulators in neurological injury through divergent yet complementary molecular pathways. Clinical-translational evidence reveals PVT1 silencing reduces cerebral infarct volume in acute ischemic stroke models by inhibiting ferroptosis via miR-214/transferrin receptor 1 (TFR1)/tumor protein p53 (TP53) axis (Lu et al., 2020). Conversely, serum exosome-derived NEAT1 promotes cerebrovascular endothelial ferroptosis in septic encephalopathy through concurrent modulation of miR-9-5p/transferrin receptor (TFRC) iron transport and glutamic-oxaloacetic transaminase 1 (GOT1) signaling pathways (Wei et al., 2022). Complementing these, hyperglycemia-exacerbated hypoxia induces Meg3-dependent ferroptosis in brain microvascular endothelia via p53/GPX4 antioxidant defense disruption (Chen et al., 2021). Collectively, these systematically mapped relationships (Table 4) demonstrate lncRNAs orchestrate ferroptosis through three convergent mechanisms: (1) iron homeostasis regulation (PVT1/TFR1, NEAT1/TFRC), (2) antioxidant pathway disruption (Meg3/GPX4), and (3) cellular compartmental specificity (neuronal vs. endothelial targeting), highlighting their roles as tissue-contextual arbiters of iron-dependent cell death.

**TABLE 4:** Long non-coding RNAs (LncRNA)-driven neuronal ferroptosis: targets and mechanisms.

LncRNA	Disease model	Expression	Molecular axis	Functional outcome	References
PVT1	Acute ischemic stroke	↓	PVT1↓→↑miR-214 →↓TFR1/↓p53	Inhibits ferroptosis through miR-214-mediated suppression of TFR1/p53	Wei et al., 2022
NEAT1	SAE	↑	NEAT1↑→↓miR-9-5p →↑TFRC/↑GOT1	Exosomal NEAT1 drives ferroptosis via miR-9-5p sponging and TFRC/GOT1 axis activation	Chen et al., 2021
Meg3	Diabetic perioperative stroke	↑	Meg3↑→ p53↑→↓GPX4	Mediates p53-dependent ferroptosis via GPX4 inhibition in diabetic brain ischemic damage	Hitomi et al., 2008

SAE, sepsis-associated encephalopathy; TFR1, transferrin receptor 1; GPX4, glutathione peroxidase 4; TFRC, transferrin receptor; GOT1, glutamic-oxaloacetic transaminase 1; miR, microRNA;↑, upregulation; ↓, downregulation.

### 2.5 Necroptosis

Necroptosis represents a caspase-independent programmed cell death pathway defined by cytoplasmic swelling, plasma membrane rupture, and inflammatory mediator release. Its molecular cascade involves hierarchical activation: RIPK1-RIPK3 interaction initiates necrosome assembly, leading to RIPK3 autophosphorylation and MLKL recruitment (Hitomi et al., 2008; Tran et al., 2024). Subsequent MLKL phosphorylation induces oligomerization and plasma membrane translocation, forming pores that disrupt ionic homeostasis—the hallmark execution event. Beyond this canonical axis, RIPK3 orchestrates alternative signaling via mitochondrial phosphatase PGAM5, which stabilizes RIPK1/MLKL while activating CAMKII isoforms and ROS cascades (Cho et al., 2009; Prasad Panda et al., 2023; Zhan et al., 2022). This cross-compartmental signaling amplifies necroptotic responses, demonstrating the pathway’s capacity for diversification beyond the core RIPK1-RIPK3-MLKL axis while retaining membrane permeabilization as the terminal effector mechanism.

Necroptosis drives neuroinflammation through cytokine release and inflammasome activation, serving as a pathogenic mechanism in multiple sclerosis (MS), amyotrophic lateral sclerosis (ALS), Parkinson’s disease (PD), and Alzheimer’s disease (AD) (Richard and Mousa, 2022; Yuan et al., 2019). Mechanistic studies establish RIPK1-driven necroptosis as clinically relevant in neurological pathologies: In Alzheimer’s disease models, pharmacological inhibition of RIPK1 by necrostatin-1 attenuates tau hyperphosphorylation and Aβ-induced neuroinflammation while rescuing cognitive impairment in APP/PS1 mice (Yang et al., 2017). In parallel, stroke models reveal hypoxia-induced RIPK1/RIPK3/MLKL pathway activation driving neuronal damage, where necrostatin-1 intervention improves neurological recovery in ischemic and hemorrhagic stroke by blocking necroptotic signaling (Chen et al., 2018; Shen et al., 2017). Genetic ablation studies confirm RIP1/RIP3 deficiency protects against acute ischemic injury through dual suppression of necroptosis and neuroinflammation (Zhang et al., 2020), collectively establishing necroptosis inhibition as a promising therapeutic strategy for acute and chronic neurodegenerative conditions.

Notably, cancer biology reveals conserved lncRNA-mediated necroptosis regulation relevant to neurological contexts: Colon cancer studies identify a 6-lncRNA signature modulating TNF-α/NF-κB-driven necroptosis (Liu et al., 2022), gastric adenocarcinoma research shows 12 lncRNAs regulating caspase-8/FADD-mediated death (Luo et al., 2022), and lung adenocarcinoma models reveal 7 prognostic lncRNAs linked to MLKL ubiquitination (Lu et al., 2022). Critically, shared regulatory architecture emerges with identical necroptotic effectors (RIPK1, caspase-8, MLKL) in cancer and neurodegeneration (Najafov et al., 2019; Yuan et al., 2019), lncRNA bridging via cancer-associated regulators exhibiting CNS expression (PVT1 (Zhang et al., 2019), MALAT1 (Huang et al., 2023)), and therapeutic translatability of small-molecule inhibitors effective in both ischemic stroke (necrostatin-1 (Deng et al., 2019)) and cancer. These findings establish lncRNAs as evolutionarily conserved necroptosis modulators whose mechanistic elucidation in oncology provides direct insights for developing targeted neurotherapeutics.

### 2.6 Cuproptosis

Cuproptosis—a copper-dependent cell death pathway mediated by mitochondrial proteostasis collapse via copper binding to lipoylated enzymes (e.g., dihydrolipoamide S-acetyltransferase, DLAT)—involves oligomerization of lipoylated proteins and iron-sulfur cluster degradation, culminating in membrane damage via proteotoxic mechanisms (Deng et al., 2024; Tsvetkov et al., 2022). Dysregulated cuproptosis contributes to pathological manifestations in specific oncological contexts. In malignancies, dysregulation of ATP7A/B transporters mediates chemotherapy resistance, as evidenced by established prognostic models: ATP7B downregulation correlates with platinum resistance in lung cancer, while ATP7A features in cuproptosis-related gene signatures for hepatocellular carcinoma clinical stratification (Jawed and Bhatti, 2024; Shao et al., 2023). Meanwhile, in Alzheimer’s pathology, nitration modifications of β-amyloid peptides attenuate copper-mediated toxicity by inhibiting copper binding, with these copper-amyloid complexes established as neurotoxic mediators (Zhao et al., 2019). Elucidating CNS-specific cuproptosis mechanisms may reveal multipronged therapeutic opportunities, including copper-chelating agents modulating copper homeostasis (Jiang et al., 2022), metabolic modulators targeting copper-associated pathways (Gao and Zhang, 2023; Kong and Sun, 2023), and exploratory lncRNA-based interventions guided by hepatic malignancy-identified cuproptosis regulatory signatures (Mao et al., 2023). This mechanistic understanding facilitates neuroprotective intervention development for neurological disorders.

Cuproptosis signatures demonstrate significant translational potential in neuro-oncology. Studies by Zhu et al. (2024) develop an 18-gene cluster predicting low-grade glioma (LGG) survival and immunotherapy response, while subsequent research establishes lncRNA-based prognostic models incorporating 9–10 cuproptosis-associated transcripts—including functional regulators like LEF1-AS1 that suppress glioma proliferation and invasion (Chen et al., 2023; Jin et al., 2023; Wang et al., 2022). Bioinformatic analyses of Alzheimer’s datasets reveal dysregulated cuproptosis-associated genes (MTF1, NFE2L2, GLS) and lncRNAs (LY86-AS1, MIR7-3HG) (Zeng et al., 2024). These shared molecular perturbations suggest potential conservation of copper-dependent death pathways across CNS disorders, though mechanistic validation in other neuropathologies remains required. The convergence of cuproptosis-associated signatures in neuropathologies—spanning oncogenesis to proteinopathy—highlights its role as a fundamental cell death mechanism whose tissue-specific regulatory networks require prioritized validation in human neuronal models.

## 3 Conclusion

Long non-coding RNAs critically coordinate neural cell death in neurological disorders through multimodal regulatory networks, concurrently modulating caspase-dependent apoptosis and non-apoptotic pathways—including ferroptosis, necroptosis, and cuproptosis—via distinct molecular interactions. Persistent knowledge gaps remain regarding PCD crosstalk (e.g., apoptosis-necroptosis interplay) and context-dependent hierarchical control in CNS pathophysiology (Bertheloot et al., 2021; Ciftci et al., 2024; Malireddi et al., 2019). Notably, emerging evidence reveals functional diversification beyond canonical non-coding roles: a subset of lncRNAs encodes regulatory peptides that orchestrate cellular functions through ribosome-independent mechanisms. This creates a molecular duality that, coupled with precise spatiotemporal expression patterns, positions these molecules as ideal candidates for biomarker development and therapeutic targeting (Bakhti and Latifi-Navid, 2022; Wu et al., 2020; Xing et al., 2021). Future research must address three critical imperatives to leverage this potential: systematically mapping lncRNA-peptide interactomes in neural death pathways using advanced crosslinking immunoprecipitation; resolving region-specific regulatory networks via integrated single-cell/spatial omics; and validating evolutionary conservation of functional motifs through cross-species models. Achieving this will enable CRISPR-engineered organoid platforms to accelerate lncRNA-targeted neurotherapeutic translation.
